# Interfacial engineering with chemical bath deposition for high-performance HgTe quantum dot-based short-wave infrared photodetectors

**DOI:** 10.1186/s40580-025-00519-9

**Published:** 2025-10-29

**Authors:** Haoran Chen, Yuwei Guo, Yulia V. Kuznetsova, Kseniia A. Sergeeva, Arsenii S. Portniagin, Xie He, Hui Yu, Andrey L. Rogach, Ni Zhao

**Affiliations:** 1 Department of Electronic Engineering, The Chinese University of Hong Kong, Hong Kong SAR 999077 Shatin, New Territories, People’s Republic of China; 2 Department of Materials Science and Engineering and Centre for Functional Photonics (CFP), City University of Hong Kong, 83 Tat Chee Avenue, Kowloon, 999077 Hong Kong SAR People’s Republic of China

**Keywords:** HgTe quantum dots, Short-wave infrared phototransistor, Chemical bath deposition, Interfacial engineering, CdS layer

## Abstract

Colloidal quantum dot-based short-wave infrared (SWIR) photodetectors are often limited by surface traps and high noise levels at room temperature. In this work, we present a low-temperature chemical bath deposition (CBD) strategy to grow a heterojunction passivation layer on HgTe quantum dot (QD) photoactive layers, enabling high-performance SWIR photodetection at room temperature. The CBD process achieves interfacial modification through a dual mechanism: sulfur ions penetrate the HgTe QD surface to form an Hg-S bonded interfacial region while simultaneously reacting with Cd^2+^ in the bath to create a CdS electron-accepting layer, resulting in a compositionally graded CdS/Hg-S/HgTe structure. The resulting interfacial improvement, coupled with energy level modification, facilitates carrier separation and passivates surface defects, thus simultaneously enhancing the responsivity and reducing noise current of photodetectors. As a result, the phototransistor based on the CdS/HgTe photoactive layer demonstrates a high room-temperature specific detectivity of 4.43 × 10^11^ Jones at 1550 nm and maintains detectivity around 10^10^ Jones at extended wavelengths up to 2500 nm. These results underscore the importance of interfacial engineering in colloidal QDs-based photodetectors and demonstrate CBD as a scalable, silicon-compatible passivation approach for achieving cryogen-free SWIR optoelectronic devices.

**Graphical abstract:**

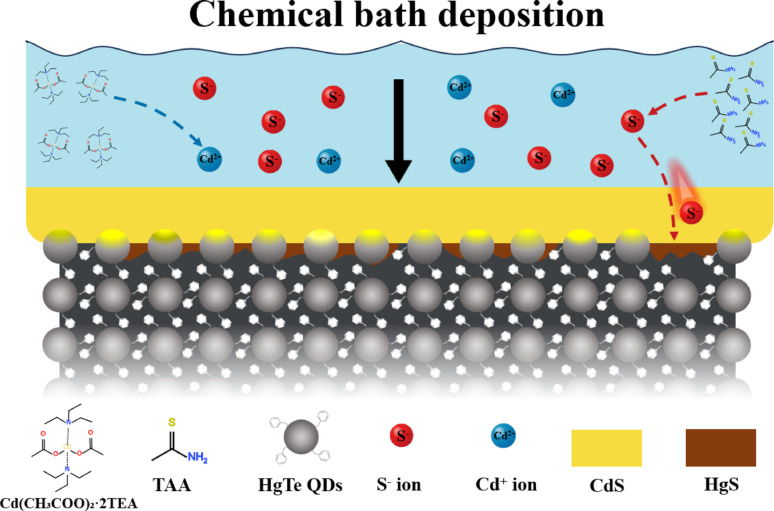

**Supplementary Information:**

The online version contains supplementary material available at 10.1186/s40580-025-00519-9.

## Introduction

Infrared (IR) photodetectors have attracted extensive research attention due to their wide-ranging applications in gas sensing, medical diagnostics, environmental monitoring, and military imaging systems [1–4]. Currently, the predominant materials used in IR photodetection are epitaxially grown bulk semiconductors such as mercury cadmium telluride (HgCdTe), indium antimonide (InSb), and type-Ⅱ superlattices (such as InAs/GaSb), which possess excellent detection capabilities. However, these materials require complex and high-cost fabrication processes [5–7], cryogenic cooling operation [5, 8], and exhibit poor compatibility with silicon integrated circuits [5, 8], thus limiting their scalability and applicability. In contrast, colloidal quantum dots (QDs), with their solution processability, size-tunable bandgap, and compatibility with flexible substrates, present a promising, low-cost alternative for infrared photodetectors that operate at room temperature [9].

Among these QD materials, mercury telluride (HgTe) quantum dots have emerged as a promising candidate, owing to the zero-gap nature of this bulk semiconductor, which enables tunable absorption extending deeply into the IR region [10–16]. Additionally, HgTe QDs exhibit inter-band transitions, tunable Fermi levels, and excellent adaptability to diverse device configurations, making them highly attractive for integrated IR photodetection systems [1]. Despite having these advantages, the performance of HgTe QD-based devices is still largely hindered by several intrinsic limitations. Due to their high surface-to-volume ratio and the presence of organic ligands, HgTe QDs tend to suffer from a high density of surface defects and poor carrier mobility [17]. These issues lead to severe interfacial recombination, high dark current, and low detectivity. As a result, achieving high-performance IR photodetection at room temperature remains challenging.

To address these challenges, interfacial engineering has emerged as a crucial strategy for improving photo-carrier extraction and suppressing non-radiative recombination [18–20]. Conventional methods to form charge transport layers or shell layers (such as atomic layer deposition or in-situ shell growth) are effective but often require high temperature, which is detrimental to organic ligand passivation of HgTe QDs [21]. On the other hand, chemical bath deposition (CBD) provides a milder, low-temperature alternative, enabling the deposition of uniform and conformal semiconductor layers [22–24].

In this work, we demonstrate a CBD process for forming a compositionally graded cadmium sulfide (CdS) capping layer on a HgTe QD active layer within an IR phototransistor [25]. Specifically, the anhydrous, low-temperature CBD process induces a composition interfacial modification, resulting in a CdS/Hg-S/HgTe heterostructure at the surface of the QD film. This structure significantly reduces trap states and suppresses recombination losses. Additionally, it functions as a type-II heterojunction, further facilitating spatial carrier separation. The synergistic effects achieved by this approach enhance the room-temperature IR detection performance of the device by simultaneously increasing responsivity and reducing noise current, leading to an over 300-fold improvement in detectivity.

## Materials and methods

### Materials

Mercury(II) chloride (HgCl₂, Sigma–Aldrich, ACS Reagent), elemental tellurium (Te, Sigma–Aldrich, 99%), benzyl mercaptan (BMT, Acros, 99%), trioctylphosphine (TOP, Sigma–Aldrich, 97%), tripropylamine (TPA, Sigma–Aldrich, 98%), cadmium(II) acetate (Cd(CH₃COO)₂, TCI, 98%), thioacetamide (TAA, Alfa Aesar, 99%), triethylamine (TEA, Sigma–Aldrich, 99.5%), isopropanol (IPA, Sigma–Aldrich, 99.5%), N, N-dimethylformamide (DMF, Sigma–Aldrich, 99.8%), ethyl acetate (EA, Anaqua, ACS), n-hexane (HX, Anaqua, 99%, HPLC), and high-purity argon gas (Ar, 99.999%) were all used as received without further purification.

### Synthesis of HgTe QDs

HgTe QDs were synthesized using a hot injection method. In a three-neck flask, 1.5 mmol of HgCl₂ was dissolved in DMF alongside with BMT and TPA (Hg: BMT: TPA molar ratio was 1:4:4). The solution was degassed for 15 min at room temperature, and heated to 85 °C under Ar flow, maintaining stability without any precipitation. After temperature stabilization, 0.9 ml of 1 M [TOP-Te] was injected, and the particle growth continued for 2 min before stopping the reaction by rapidly cooling in an ice bath. For further use for analysis and device fabrication, the crude solution was precipitated by adding the EA and HX (sample: EA: HX = 1:1:2 volume ratio). Then the precipitate was collected by centrifugation at 4200 rpm for 5 min. Finally, the sediment was dried by Ar flow and redissolved in DMF up to concentration of 30 mg/ml.

1 M [TOP-Te] precursor synthesis: 30 mmol of elemental tellurium were mixed with 30 mL of TOP in a three-neck flask. The flask was degassed at 120 °C for 1 h, then filled with high-purity Ar and heated to 250 °C for 3 h.

### Device fabrication

Interdigitated drain–source gold electrodes were fabricated on silicon wafer (with ~ 300 nm SiO_2_ layer) substrates via standard photolithography, followed by thermal evaporation and a subsequent lift-off process. The channel length and width of the electrodes were 10 μm and 1 mm, respectively, comprising 15 repeated unit pairs. The total optical sensing area of all devices reported in this work was 1.5 × 10^−3^ cm^2^ [26]. The substrate’s treatment in ozone (30 min) and the active layer deposition were conducted in a nitrogen-filled glovebox. The pristine HgTe QDs solution was filtered through a 0.45 μm hydrophobic pore filter to remove any aggregated particles before to film deposition. For QD layer deposition, 30–40 µL of the purified HgTe QD solution was drop-cast onto the pre-patterned silicon substrate and spin-coated at 1600 rpm for 40 s. The resulting film was then annealed on a hot plate at 55 °C for 5 min and rapidly cooled to room temperature. This deposition procedure was repeated five times to achieve an active layer thickness of approximately 80 nm. The fabricated devices were subsequently subjected to CBD processing.

### Chemical bath deposition of cds

Since conventional aqueous CBD is potentially harmful for water-and oxygen-sensitive HgTe QD films, we adopted an anhydrous, low-temperature, short-duration CBD that preserves the QD surface while enabling interfacial passivation. Specifically, the modified version of aqueous CBD was used for CdS deposition [22–24]. In present work, 13.8 mg of cadmium(II) acetate and 250.5 µL of triethylamine were dissolved in 5 mL of IPA under stirring and gently heated to 55 °C. Separately, 9 mg of thioacetamide was dissolved in another 5 mL of IPA. The two precursor solutions were then combined and maintained at 55 °C. The HgTe QDs-coated silicon wafer substrates were vertically immersed into the resulting bath solution and held at 55 °C for 10 min to deposit the CdS layer, which intentionally minimized QD sintering.

### Materials and device characterization

X-ray and ultra-violet photoemission spectra (XPS and UPS, respectively) were measured on a ThermoFisher ESCALAB250 X-ray photoelectron spectrometer with AlKα source gun (up to 1100 eV). The data were collected from a uniform area with a diameter of 500 μm and was used to analyze the Hg*4f*,Te*3d*,Cd*3d*, S*2p *core level spectra with an energy resolution of ΔE = 0.1 eV. A Helium Ⅰ source (21.22 eV) was used to perform UPS measurements. The C*1s* peak at 284.8 eV was used as a reference. XPS data analysis involved the background normalizing to a Shirley function and fitting of the experimental spectra using a combination of Voigt-shaped peaks. The following rules were used for fitting: (i) every type of bond had two components due to spin-orbit splitting (4.05 eV for Hg*4f*_7/2_ and Hg*4f*_5/2_, 10.39 eV for Te*3d*_5/2_ and Te*3d*_3/2_, 6.74 eV for Cd*3d*_5/2_ and Cd*3d*_3/2_, 1.18 eV for S*2p*_3/2_ and S*2p*_1/2_); (ii) the area ratio of splitting lines was kept as 3:2 for *3d*_5/2_:*3d*_3/2_ and *4f*_7/2_:*4f*_5/2_ doublets, and as 2:1 for *2p*_3/2_:*2p*_1/2_ doublet; (iii) peaks width (FWHM) for components with one doublet was fixed. The work function value, W_F_, was determined using the formula W_F_ = hυ – E_cut off_ – PB, where E_cutoff_ is the secondary electron cut-off energy (in binding energy units) and PB is the polarization bias (5 V). The position of the valence band maximum (VBM, E_VBM_) was determined using the formula E_VBM_ − E_f_ = hυ − KE_VB_, where E_f_ is the Fermi energy, hυ is the energy of the He I source (21.2 eV), and KE_VB_ is the maximum kinetic energy of photoelectrons.

The optical absorption of CdS in a film was measured from 200 to 600 nm using a UV-3600 spectrophotometer (Shimadzu). The optical absorption of HgTe QDs was determined by Fourier transform infrared spectroscopy in attenuated total reflection mode (ATR-FTIR) on a Thermo Scientific Nicolette iS50 FTIR Spectrometer in the wavenumber range of 1600–8000 1/cm.

The morphological and compositional characterizations of the films were performed using a scanning electron microscope (SEM, ZEISS Sigma 360, Germany), depth-profiled X-ray photoelectron spectroscopy (XPS, Thermo Scientific K-Alpha, USA), and atomic force microscopy (AFM, Bruker Dimension Icon, Germany).

Optoelectrical performance of the devices was measured using a Keithley 2612 Source Meter, with illumination by a Newport LQD1550E laser (5 mW, 1550 nm). The current noise spectral density was measured using a fast Fourier transform (FFT) spectrum analyzer (SR760) under dark conditions, following 10 min of electrical biasing. Devices were battery-powered and shielded within an electromagnetically isolated enclosure. Measurements were conducted in dBVrms using Hanning and Blackman–Harris windows, AC coupling, and averaged over 1000 acquisitions.

Transient photocurrent measurements were carried out by modulating the Newport LQD1550E laser at 11 Hz with an Agilent 33210 A function generator. The resulting photocurrent was amplified by a Femto DHPCA-100 high-speed current amplifier and recorded using a Tektronix TDS 3014 C digital oscilloscope.

The light intensity dependent-responsivity was calibrated with the same setup using several near infrared absorptive filters. Wavelength-dependent detectivity measurements were performed under monochromatic illumination, generated by focusing the output of a 250 W quartz tungsten-halogen lamp through a Newport 74125 Oriel Cornerstone 260 1/4 m monochromator. The filtered light, passed through a 1500 nm long-pass filter, was focused onto the device surface via a pair of CaF₂ lenses. The incident optical power was calibrated using a Hamamatsu P5968-200 InSb photodetector operated at 80 K under liquid nitrogen cooling.

## Results and discussion

**Fig. 1:**
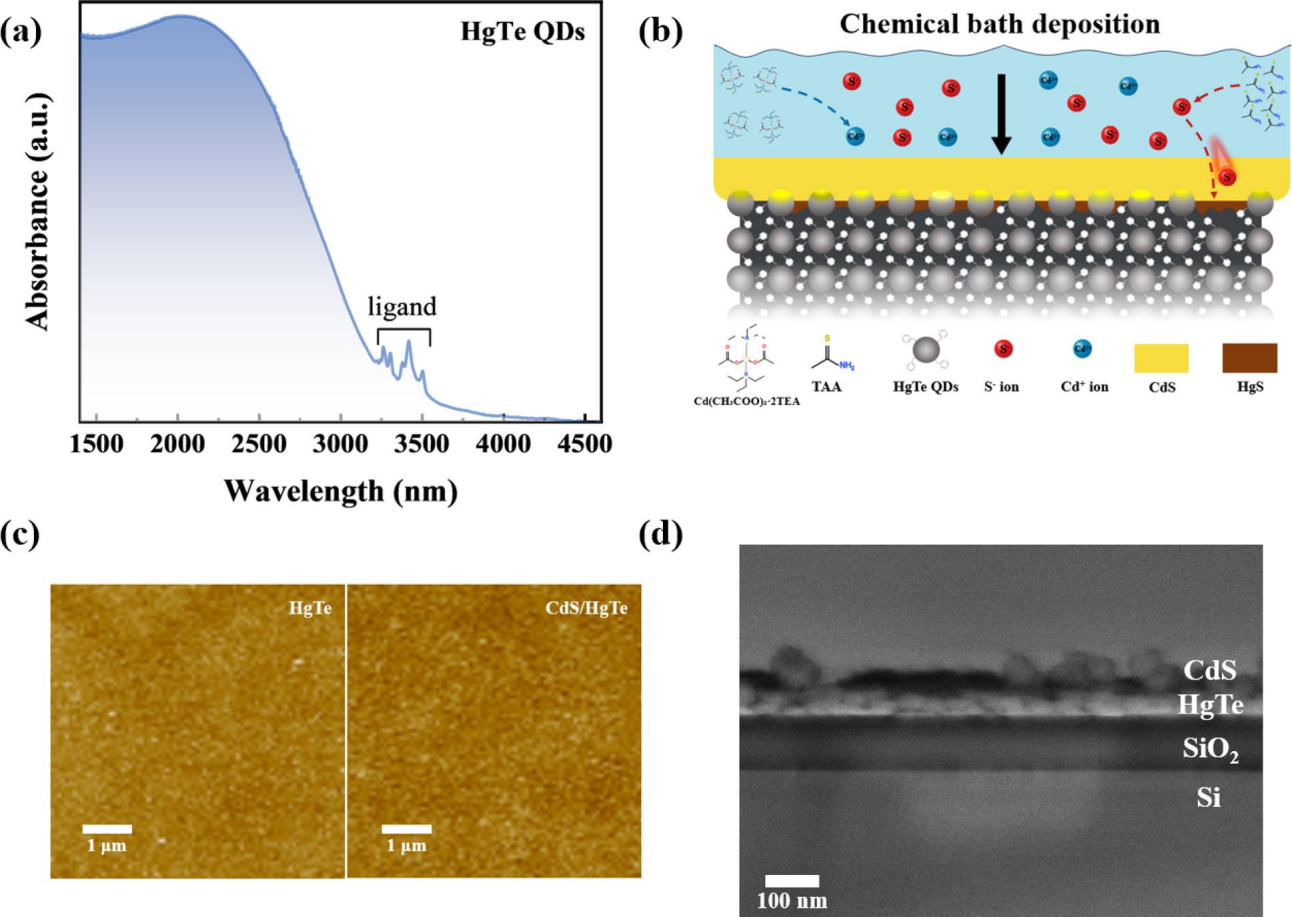
**a** Absorption spectrum of HgTe QDs.** b** Schematic illustration of the CBD process and the interfacial modification mechanism in HgTe QD thin films.** c** Atomic force microscopy images of HgTe QD films and CdS/HgTe heterojunction films. **d** Cross-sectional SEM image of the CdS/HgTe thin film

BMT-capped HgTe QDs exhibited a distinct exciton feature at approximately 2300 nm, with absorption extending up to 3000 nm (Fig. 1a). After five cycles of spin casting, a uniform QD film with a surface roughness (R_a_) of 8.99 nm and a thickness of around 80 nm was obtained. CdS was selected as the capping layer of the QD film due to its matching energy levels [11] and the strong bonding affinity between sulfur (S) and mercury (Hg). Traditional CBD typically requires an aqueous environment and elevated temperatures around 80 °C, which can lead to degradation and sintering of HgTe QDs [22–24]. To address these challenges, a modified CBD approach was developed in this work (Fig. 1b). By using thioacetamide as the sulfur precursor, the deposition process was carried out at a reduced temperature, minimizing the risk of QD degradation while ensuring uniform and conformal layer formation. Although the CBD method may result in lower CdS crystallinity and stoichiometric composition purity, these limitations can be mitigated by careful device design, as will be elaborated later. All reactions were conducted in an anhydrous solvent. We optimized the deposition conditions by tuning the temperature range and reaction time, identifying 55 °C for 10 min as the optimal parameters. At this temperature, sulfur is efficiently released from thioacetamide, while the reaction between cadmium and sulfur—regulated by triethylamine—proceeds gradually [27]. This controlled process ensures uniform layer formation while releasing sufficient sulfur to interact effectively with the underlying HgTe QD layer, as will be discussed in detail later.

**Fig. 2:**
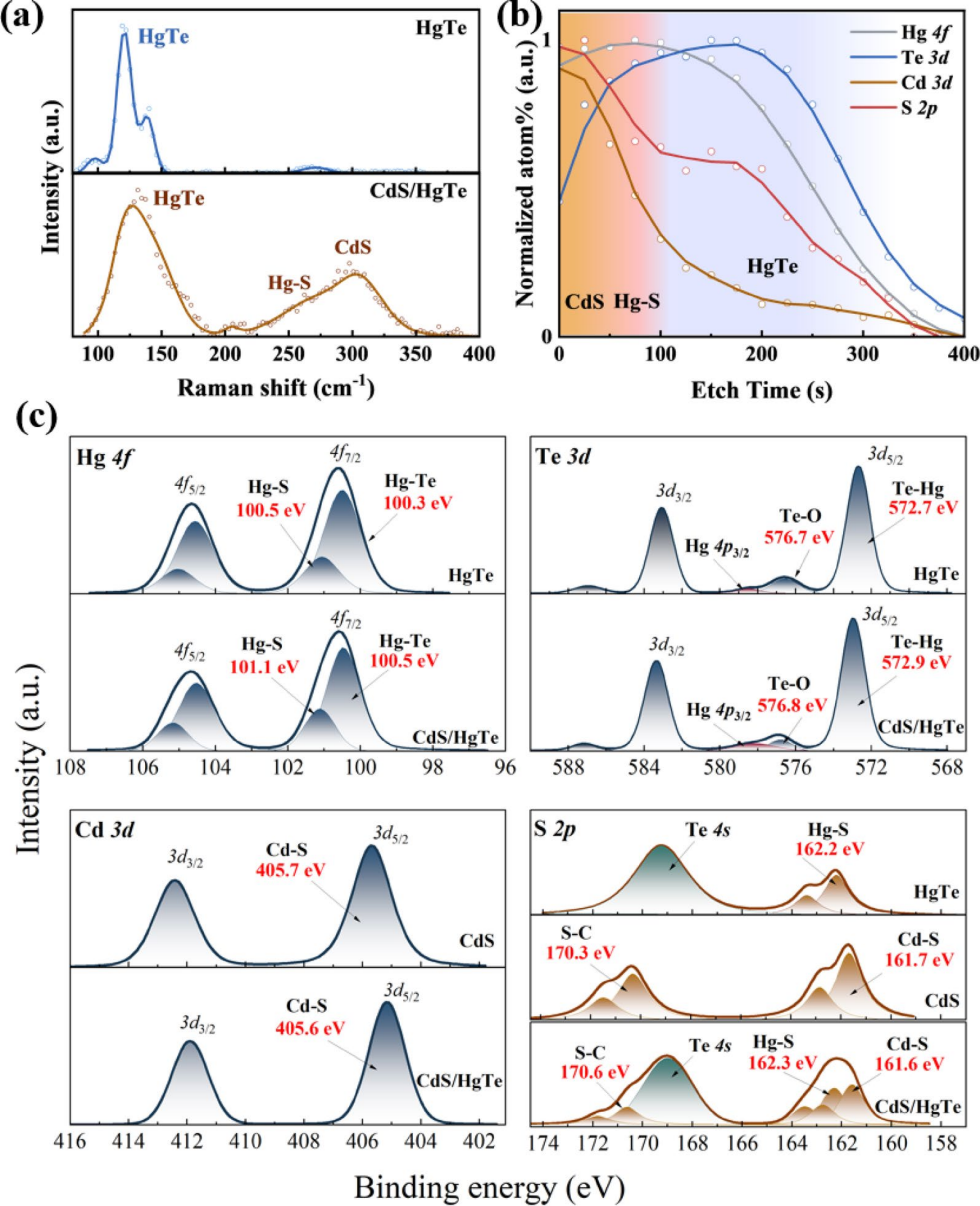
**a** Raman spectra of pristine HgTe QD and CdS/HgTe thin films.** b** Depth-profiled XPS analysis (dots) of the CdS/HgTe structure, showing elemental distribution as a function of etching time. Solid lines are given to guide the eye.** c** XPS spectra of HgTe, CdS, and CdS/HgTe films

The optimized CBD process yields a compact CdS film while preserving the structural integrity of the underlying HgTe QD layer, as evidenced by AFM images of the QD thin films before and after CBD (Fig. 1c). The height profiles (Figure S1) of both the pristine HgTe QD film and CdS/HgTe heterostructure reveal an increase in film thickness with averaged values from 80 ± 5 nm to 90 ± 5 nm after CdS deposition obtained from multiple measurements across the active layer/substrate edge. This indicates partial infiltration of CdS into the HgTe QD layer and the formation of a thin interfacial layer. A cross-sectional SEM image of the CdS/HgTe QD heterojunction deposited on a silicon wafer (Fig. 1d) show that the HgTe QDs form a continuous and uniform layer, while the CdS layer exhibits clear phase separation from the underlying HgTe layer. Furthermore, as shown in Figure S2, the absorption edge of the deposited CdS film occurs near 430 nm, significantly shifted from that of bulk CdS. The pronounced absorption tail extending up to 600 nm can be ascribed to the presence of nanocrystal aggregates and light scattering phenomena within the CdS thin film. Additionally, this extended absorption may reflect the presence of a significant number of defect states in the CdS layer. These defect states may act as long-lived traps, which beneficially modulate the photoconductive gain of the CdS/HgTe photodetectors. Next, we investigated the interface of the CdS/HgTe QD layers. Raman spectroscopy was employed to compare the HgTe QD films before and after CBD treatment. As shown in Fig. 2a, the pristine HgTe QD film exhibits four distinct peaks, corresponding to the vibrational modes of acoustical and optical phonons in HgTe QD lattice [11]. We also emphasize the presence of Hg-S chemical bonds in the QD film which is attributed to the strong attachment of BMT ligand via the thiol functional group to the Hg-rich QD surface. One has to admit that due to the similar bond lengths between in Hg-Te and Hg-S (around 2.8 Å) it is challenging to distinguish the latter in the structure of LO2 peak at 260 cm^− 1^ of Raman spectrum of HgTe QDs [28]. After the CBD process, a new Raman peak emerges at 305 cm^− 1^, which is characteristic of CdS, accompanied by a notable increase in the Hg-S signal from 200 to 270 cm^− 1^ and a concurrent reduction in the HgTe peak intensity [29]. These spectral changes, specifically the emergence of the bands at 205, 235 and 257 cm^− 1^, correlate well with the literature data on multi phonon scattering in nanocrystalline CdS [30, 31]. Moreover, the broadening of the bands at 250–260 cm^− 1^ suggests that sulfur ions from the bath have infiltrated the HgTe film, resulting in substantial interfacial modification. This conclusion is further supported by depth-profiled XPS conducted via time-resolved argon ion etching (Fig. 2b). The Cd and S signals are predominantly detected at the surface; the Cd signal rapidly attenuates with increasing etching time, while the S signal exhibits a more dispersed profile, extending deeper into the film. In contrast, the Hg signal is present from the surface, whereas the Te signal is initially weak near the surface but intensifies with depth. These contrasting depth-dependent trends reveal a clear spatial separation of elements, confirming that the CdS layer is primarily located on the top surface, while a portion of Hg-S also forms at the interface, contributing to the interfacial modification of the underlying HgTe QDs. This conclusion is further supported by XPS comparative analysis on the bare HgTe QD film, CdS film, as well as their heterojunction structure. As shown in Fig. 2c, the binding energies of the Hg *4f*, Te *3d*, and Cd *3d* peaks remain unchanged after hybridization, indicating that the CBD of CdS does not change the local chemical environments of Hg and Te [17]. The consistent and weak intensity of oxidized Te component (Te-O) suggests the resistance of HgTe QDs to oxidation during CBD and device fabrication. The high resolution XPS of HgTe evidences on the presence of two Hg species: core Hg bound to Te (Hg-Te) and surface Hg bound to thiol-containing ligands (Hg-S). The increased relative area of the Hg-S component in the hybrid CdS/HgTe structure indicates effective passivation of free surface Hg ions by sulfur ions during CBD. The S *2p* spectrum exhibits a more complex profile combining different sulfur species. The fitting of XPS data on CdS film evidences on the presence of S-Cd (S bound to Cd, S *2p*_3/2_ at 161.7 eV) [32] and organic sulfur compound S-C (S bound to C, S 2*p*_3/2_ at 170.3 eV) associated with unreacted thioacetamide. The HgTe sample shows a signal (S *2p*_3/2_ at 162.2 eV) corresponding to sulfur from thiol groups attached to mercury (Hg-S). A broad and intense peak at 169 eV corresponds to the Te *4s* signal in the HgTe sample. The sulfur signal in the CdS/HgTe heterojunction structure represents a combination of all mentioned sulfur species. A subtle peak shift of Hg-S component confirms that unpassivated Hg^2+^ on the HgTe surface bond to S^2−^ during the CBD, effectively eliminating dangling bonds. Figure 2b illustrates such composition gradient at the CBD-processed CdS/HgTe QD interface.

**Fig. 3:**
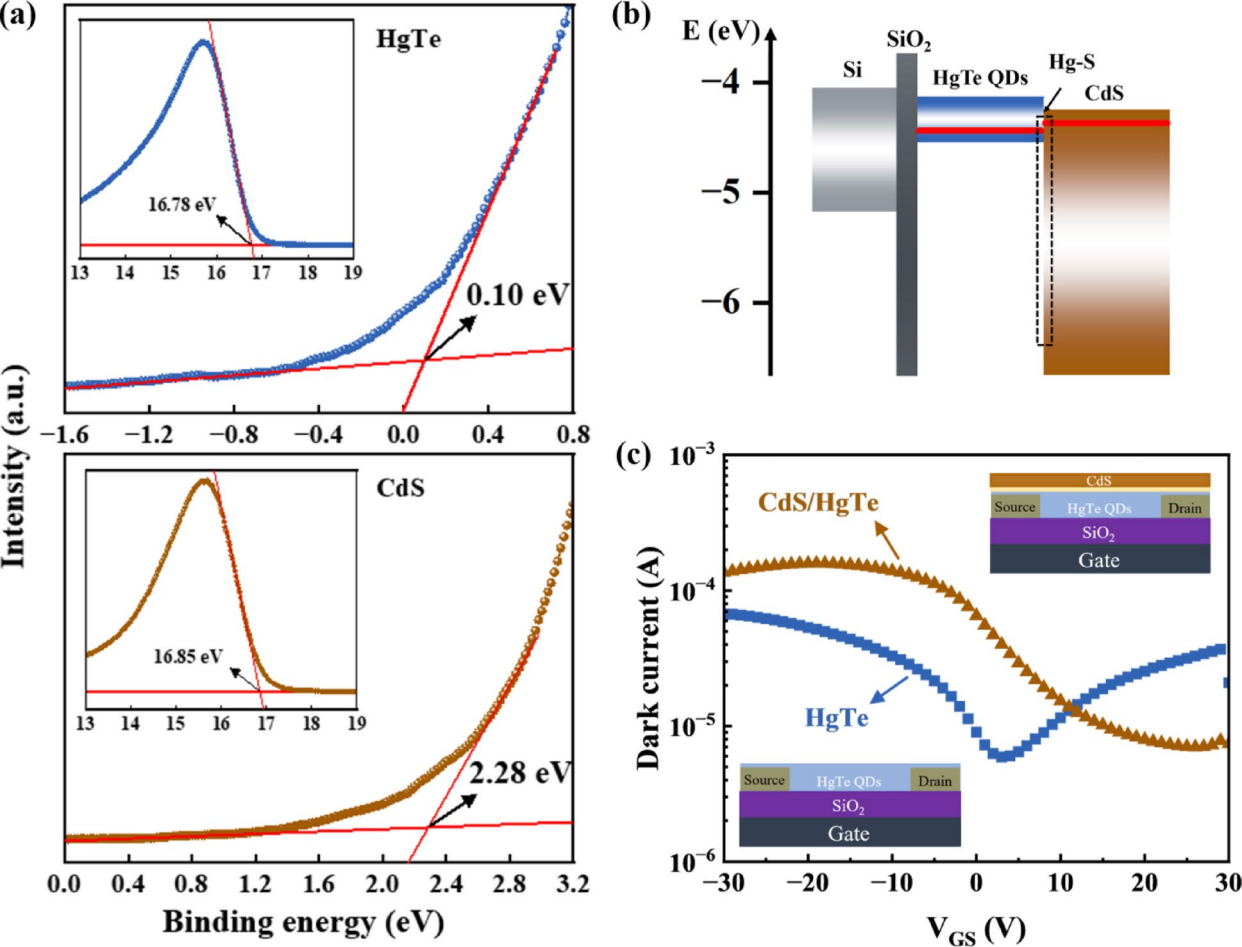
**a** UPS spectra of HgTe and CdS films.** b** Energy band diagram of the CdS/HgTe structure.** c** Typical transfer characteristics of HgTe QDs- and CdS/HgTe-based phototransistors measured in the dark under forward V_GS_ sweep from + 30 to − 30 V at a fixed V_DS_ of − 20 V, and schematic illustration of the phototransistor architecture

To investigate the energy-level alignment at the interface, UPS was conducted on bare HgTe and CdS films, as presented in Fig. 3a. The valence band maxima of HgTe and CdS were found to lie 0.10 and 2.28 eV below the Fermi level, respectively. The work functions of HgTe and CdS were calculated to be 4.44 and 4.37 eV, respectively. Based on the bandgap value calculated from the absorption spectrum, the conduction band minimum values are estimated to be 4.13 and 4.25 eV for HgTe and CdS, respectively. Combining the band information of each material, the schematic diagram of the heterojunction is provided in Fig. 3b, showing a typical type-II p-n heterojunction. The energy levels of the Hg-S bonded interfacial region are taken from the literature [33, 34]; however, due to the ultrathin, and likely non-crystalline nature of this layer, its role in modulating electron extraction is expected to be insignificant. In this configuration, electrons are driven into the CdS electron accepting layer, while holes are blocked by the interface energy barrier and transported along the source–drain channel toward the external circuit, resulting in an enhanced photocurrent.

**Fig. 4:**
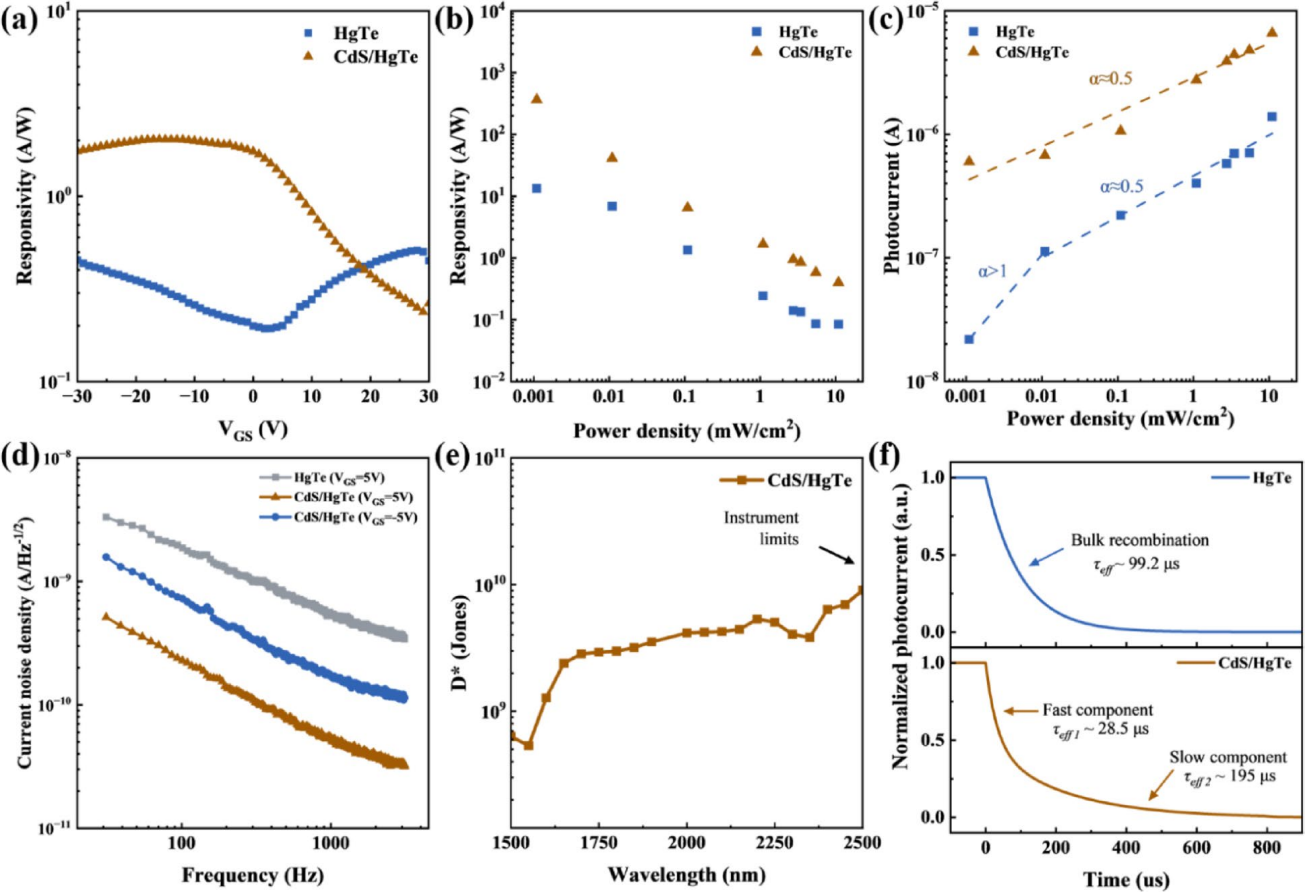
Photodetection performances of pristine HgTe and CdS/HgTe phototransistors.** a** Gate voltage-dependent responsivity measured under 1550 nm illumination at 11 mW cm^-2^,** b** Light intensity-dependent responsivity,** c** Light intensity dependent photocurrent,** d** Current noise spectral density measured at various voltage conditions,** e** CdS/HgTe phototransistor wavelength-dependent specific detectivity (at 2.5 kHz), and** f** Transient photoresponse

Utilizing the CdS/HgTe QD heterostructure, we fabricated a phototransistor, as illustrated in Fig. 3c and Figure S4. For comparative analysis of the device operation mechanism, a control phototransistor based on a bare HgTe QD layer was also fabricated under identical fabrication conditions. The typical transfer characteristics of the HgTe and CdS/HgTe phototransistors under dark conditions are shown in Fig. 3c. The fabricated pristine HgTe QDs-based phototransistor exhibits a typical ambipolar field-effect behavior with hole and electron mobility of 0.09 and 0.006 cm^2^ V^− 1^ s^− 1^, respectively [35], and on/off current ratio ≈ 11. Upon introducing CdS layer, the CdS/HgTe heterojunction device demonstrates an enhanced conductivity with p-type mobility of 0.445 cm^2^ V^− 1^ s^− 1^ and an improved on/off ratio ≈ 22. The transition from ambipolar to unipolar transistor behavior, along with a doubled on/off ratio, confirms effective electron transfer from the HgTe QD layer to the CdS layer. This transfer suppresses electron transport in the HgTe QD layer (eliminating n-type behavior) and reduces the recombination current, thereby lowering the dark current. Meanwhile, the significantly increased hole mobility indicates a substantial reduction in trap states, likely resulting from defect passivation induced during the CBD process. Interestingly, when the cadmium precursor is removed from the CBD process, leaving only the sulfur precursor, the post-treated HgTe transistor still exhibits the same level of hole mobility as the CdS/HgTe transistor (Figure S5). This observation suggests that the dominant mechanism for the improvement in hole transport is sulfur-induced defect passivation rather than the intrinsic properties of the CdS layer. A detailed comparison of the hysteresis and gate leakage currents is shown in Figure S6 of the Supporting Information. The transfer curves show that the pristine HgTe transistors exhibit strong hysteresis, indicating a high density of slow trap states at the surface and interfaces. After CdS deposition by CBD, the hysteresis is reduced, suggesting that CdS effectively passivates the HgTe QDs' surface. Next, we evaluated the photodetection performances of the devices. As shown in Fig. 4a, the responsivity measured at a gate voltage of 5 V under 1550 nm laser illumination with a power density of 11 mW cm^− 2^ is significantly enhanced from 0.2 A W^− 1^ for the pristine HgTe device to 1.3 A W^− 1^ for the CdS/HgTe device. This improvement is consistent with the increased hole mobility and the efficient separation of electrons and holes facilitated by the heterostructure. As shown in Figure S5, only sulfur-containing groups were introduced to modify the QD layer properties. The S-only/HgTe device exhibits improved hole transport, as evidenced by the p-type current reaching a level comparable to that of the CdS/HgTe device. In contrast, the modification of the n-type regime is much weaker in the S-only/HgTe device than in the CdS/HgTe device. Moreover, the responsivity of the S-only/HgTe device is significantly lower than that of the CdS/HgTe device. These results demonstrate that Cd is critical for forming the CdS electron-accepting layer, which efficiently channels electrons away from holes and enhances photoconductive gain, thereby clarifying the importance of the Cd component in the modification. Furthermore, the performance gap between the two devices, shown in Fig. 4b, becomes even more pronounced at lower light intensities. At a light intensity of 1.1 µW cm^− 2^, the responsivity increases from 13.33 A W^− 1^ for the pristine HgTe device to 365.51 A W^− 1^ for the CdS/HgTe device. The corresponding external quantum efficiency (EQE), estimated using the relation EQE = (1240 W nm A^− 1^) R/λ is as high as ~ 29,200%, which clearly demonstrates a pronounced photoconductive gain. The photoconductive gain, arises from the efficient trap passivation and separation of electrons and holes within the CdS/HgTe heterostructure, particularly benefiting responsivity enhancement at low light intensities. Fig. 4c shows the photocurrent (*I*_ph_) as a function of light intensity (*I*), following a power-law dependence described by *I*_ph_∝*I*^α^. For both devices under high light intensities, the fitted exponent α approaches 0.5, suggesting that bimolecular recombination is the dominant loss mechanism under illumination [36]. However, at low light intensities the pristine HgTe device shows α >1, indicating the dominance of trap-assisted recombination under weak illumination [26, 36, 37]. The absence of the such regime in the CdS/HgTe device suggests the effective reduction of trap states in the QD layer.

Fig. 4d presents the current noise spectral density profiles for the devices. Both devices exhibit a distinct 1/*f* trend, characteristic of dominant flicker noise. Such noise is commonly observed in thin film transistors at low frequency and can be attributed to fluctuation in mobile carrier density and mobility. Compared to the pristine HgTe device, the CdS/HgTe structure exhibits a significant reduction in noise levels. Notably, under positive gate voltage (V_GS_ = 5), the noise density is further suppressed, highlighting the superior low-noise characteristics enabled by the interfacial modification and heterostructure. Owing to the low current noise, the detectivity (D^*^) of the CdS/HgTe heterojunction device reaches 4.43 × 10^11^ Jones at an illumination wavelength of 1550 nm under low illumination intensity (1.1 µW cm^− 2^) at a frequency of 2.5k Hz, which is significantly better than that of the pristine HgTe QD device (1.36 × 10^9^ Jones). Such remarkable increase in detectivity results from the simultaneous enhancement of responsivity and the reduction of noise. Fig. 4e presents the wavelength-dependent specific detectivity (D^*^) spectrum of the CdS/HgTe phototransistor. The device exhibits D^*^ of *around* 10^10^ Jones at a wavelength of 2500 nm with a power density of 0.34 mW cm^− 2^. Notably, the device maintains high sensitivity (D^*^ >10^10^ Jones) at room temperature across an extended spectral range up to 2500 nm.

The transient photoresponses of both devices provide further insight into the effects of interfacial modification, as illustrated in Fig. 4f and Figure S8. The decay dynamics of the normalized photocurrent were recorded following the cessation of illumination, and the effective decay time constant (τ_eff_) was extracted [38–41]. Upon the interfacial modification and the formation of the heterostructure, an additional fast decay component (~ 28.5 µs) emerges, which we attribute to the passivation of HgTe QD surface defects, which facilitates efficient charge transport and promotes bimolecular recombination. Meanwhile, the slower decay component (~ 195 µs) is significantly prolonged compared to pristine HgTe (~ 99.2 µs). This originates from long-lived traps, which may reside in the CdS layer, that sustain the photoconductive gain, whereas in the unmodified HgTe QDs-based device, a single trap-limited bulk recombination mechanism dominates [42–44]. These findings further highlight the role of sulfur ion infiltration during the CBD process in partially passivating surface trap states of the HgTe QDs and thus improving the response speed of the photodetector.

## Conclusions

In summary, we have developed a low-temperature CBD approach for the growth of a heterojunction passivation layer on HgTe QD photoactive layers, enabling high-performance SWIR photodetection at room temperature. The CBD process plays a dual role in interfacial modification: sulfur ions infiltrate the HgTe QD surface to form an ultra-thin Hg-S bonded interfacial region, while simultaneously reacting with Cd^2+^ in the CBD bath to create a thin CdS electron-accepting layer. This results in a compositionally graded CdS/Hg-S/HgTe structure. The type-II band alignment established at the CdS/HgTe interface enhances carrier separation and suppresses recombination, while the sulfur induced surface defect passivation significantly reduces noise. Benefiting from these synergistic effects of interfacial modification and heterojunction band alignment, the optimized devices achieve a detectivity of 4.43 × 10^11^ Jones at 1550 nm under low-intensity illumination (1.1 µW cm^− 2^), and maintain a detectivity around 10^10^ Jones at 2500 nm. These findings highlight the critical importance of interfacial engineering in colloidal QD-based photodetectors and establish CBD as a scalable, silicon-compatible, and cryogen-free approach for advancing next-generation SWIR optoelectronic applications.

## Supplementary Information

Below is the link to the electronic supplementary material.

Supplementary Material 1

## Data Availability

The data support the findings of this study are available from the corresponding authors upon reasonable request.
