# Supplementary material for: Interfacial engineering with chemical bath deposition for high-performance HgTe quantum dot-based short-wave infrared photodetectors
Source: Nano Converg. 2025 Oct 29;12:52. doi: 10.1186/s40580-025-00519-9 (PMC12572506; doi:10.1186/s40580-025-00519-9)
Supplement: Supplementary file 1 — Supplementary Material 1 [file 40580_2025_519_MOESM1_ESM.docx]

Supporting Information

Interfacial Engineering with Chemical Bath Deposition for High-Performance HgTe Quantum Dot-Based Short-Wave Infrared Photodetectors

*Haoran Chen^1^, Yuwei Guo^1, *^, Yulia V. Kuznetsova^2^, Kseniia A. Sergeeva^2^, Arsenii S. Portniagin^2^, Xie He^1^, Hui Yu^1^, Andrey L. Rogach^2^, Ni Zhao^1, *^*

^1^ Department of Electronic Engineering, The Chinese University of Hong Kong, Shatin, New Territories, Hong Kong SAR, People’s Republic of China.

^2^ Department of Materials Science and Engineering and Centre for Functional Photonics (CFP), City University of Hong Kong, 83 Tat Chee Avenue, Kowloon, Hong Kong SAR 999077, People’s Republic of China.

^*^ Indicates the corresponding authors.

^*^ Corresponding author: Ni Zhao

E-mail address: nzhao@ee.cuhk.edu.hk

^*^ Corresponding author: Yuwei Guo

E-mail address: yuweiguo@cuhk.edu.hk


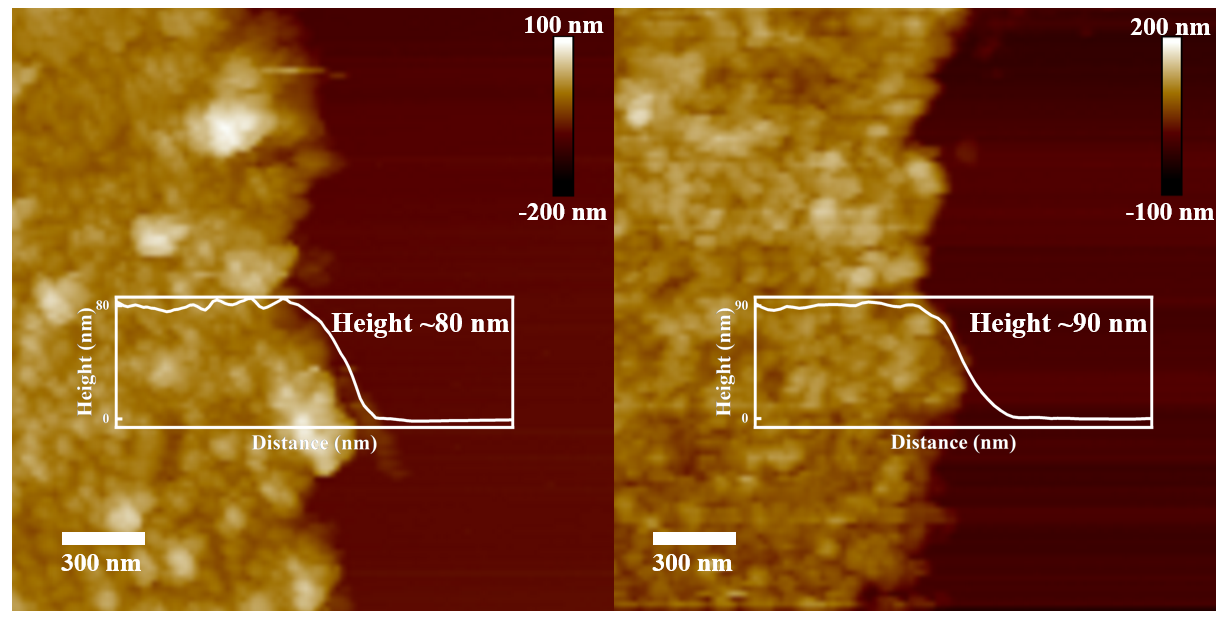


Figure S1. Atomic force microscopy images of HgTe QD films and CdS/HgTe heterojunction films, with corresponding height profiles.


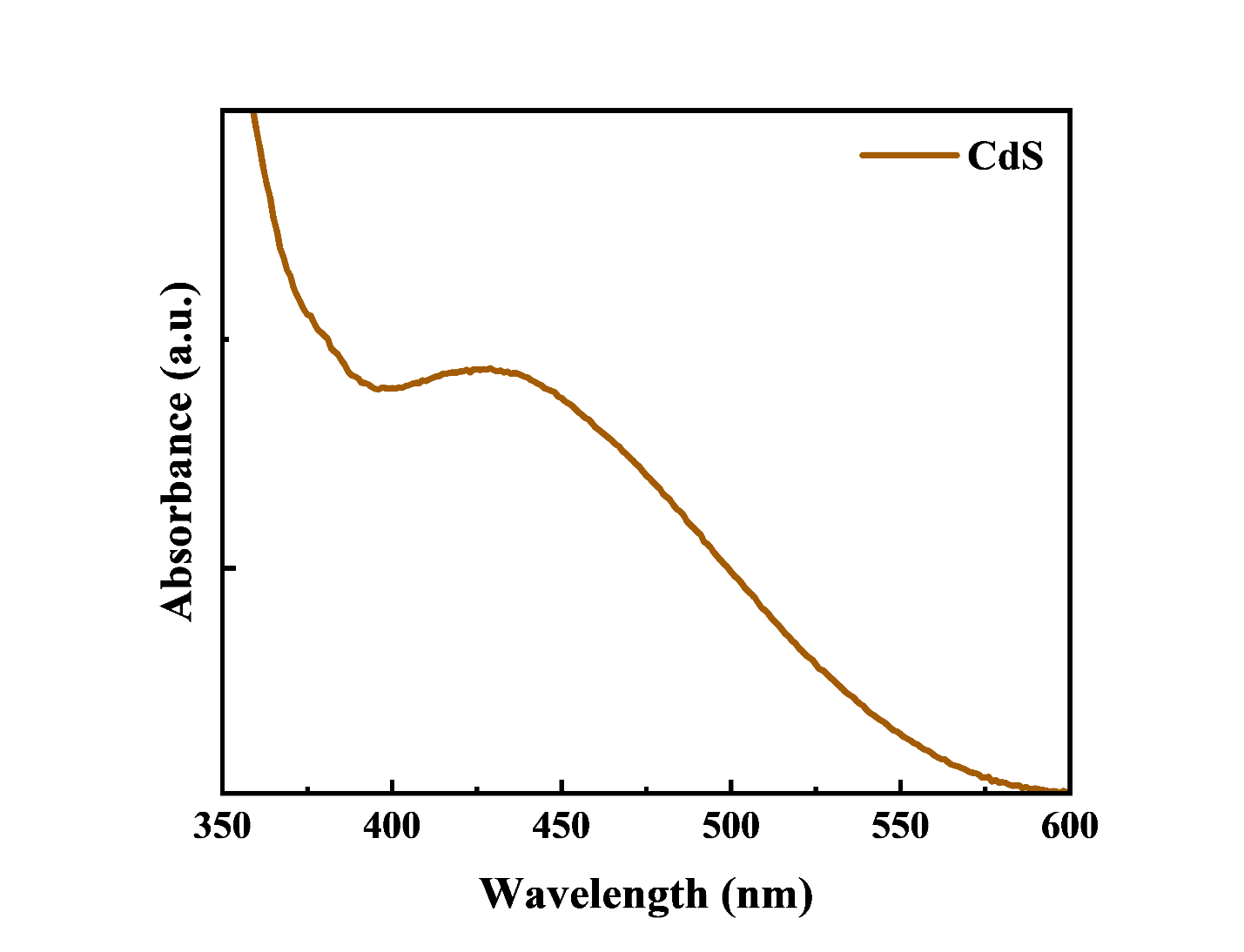


Figure S2. Absorption spectrum of CBD-deposited CdS film on glass.


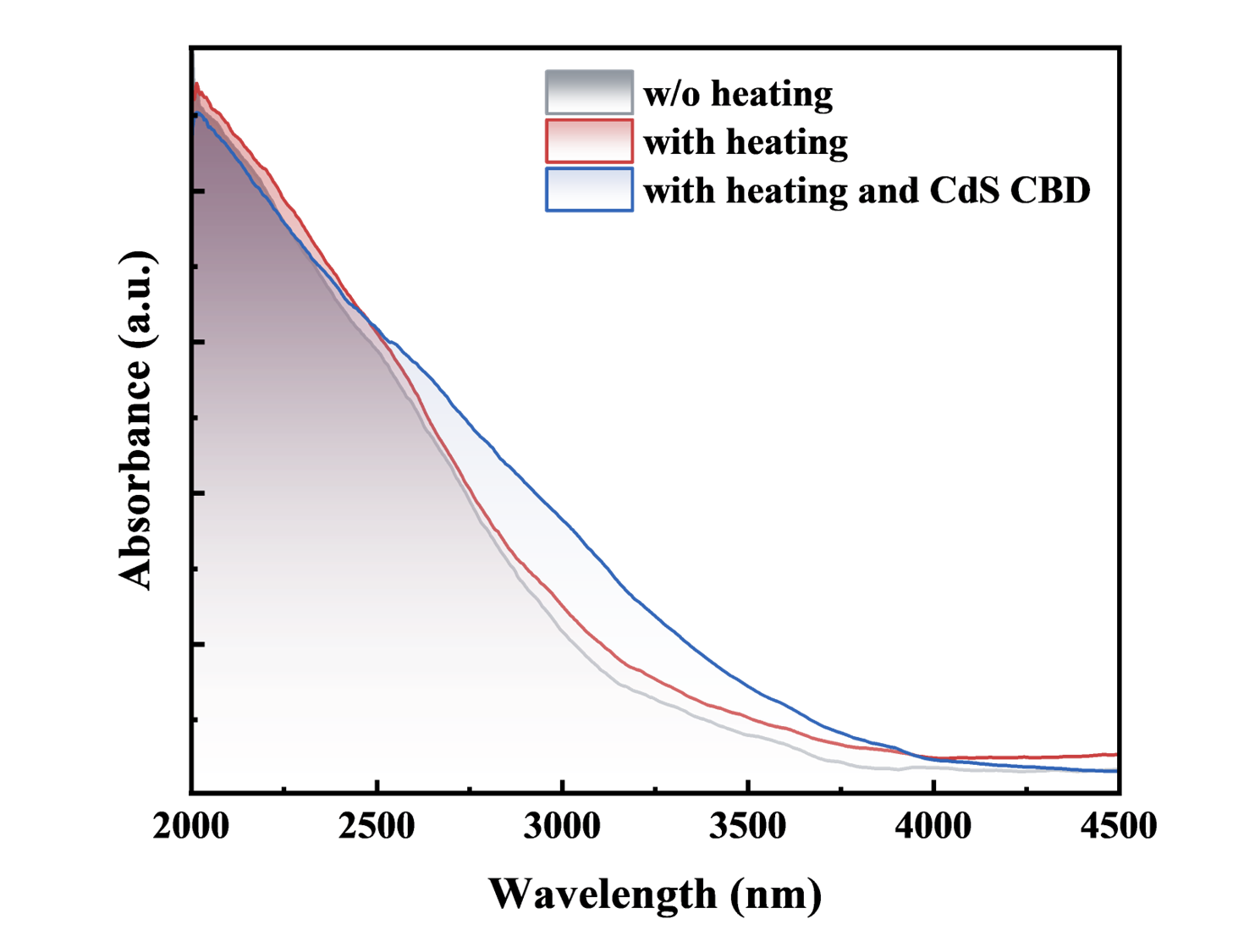


Figure S3. Absorption spectra of HgTe thin films obtained under different conditions.


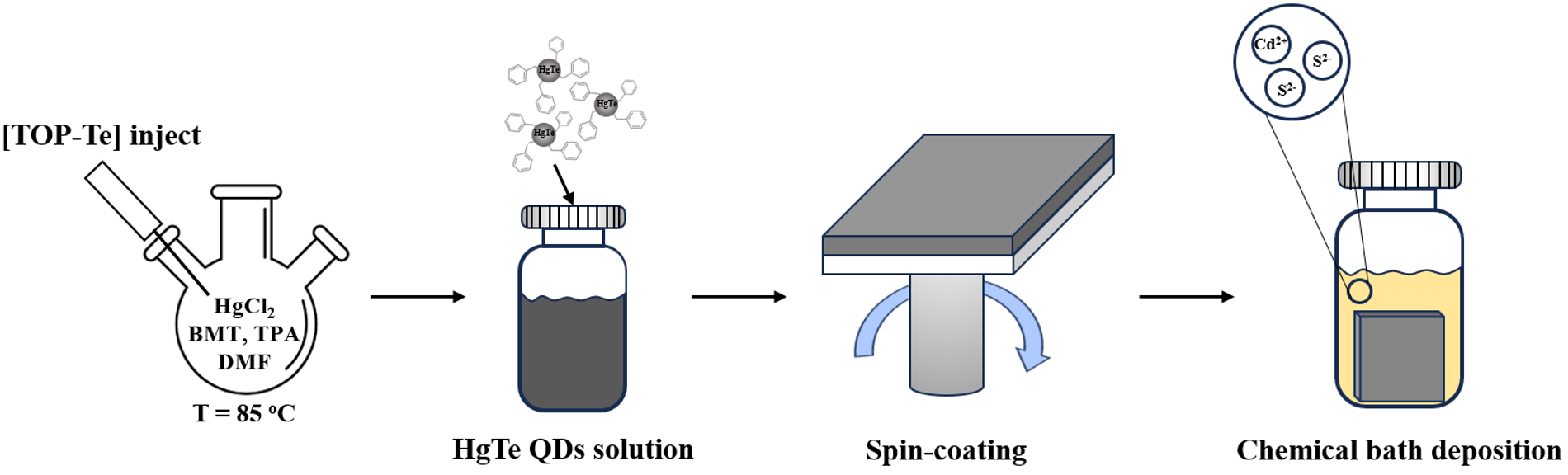


Figure S4. Schematic illustration of the synthesis of HgTe QDs and the fabrication strategy for CdS/HgTe heterojunction thin films.


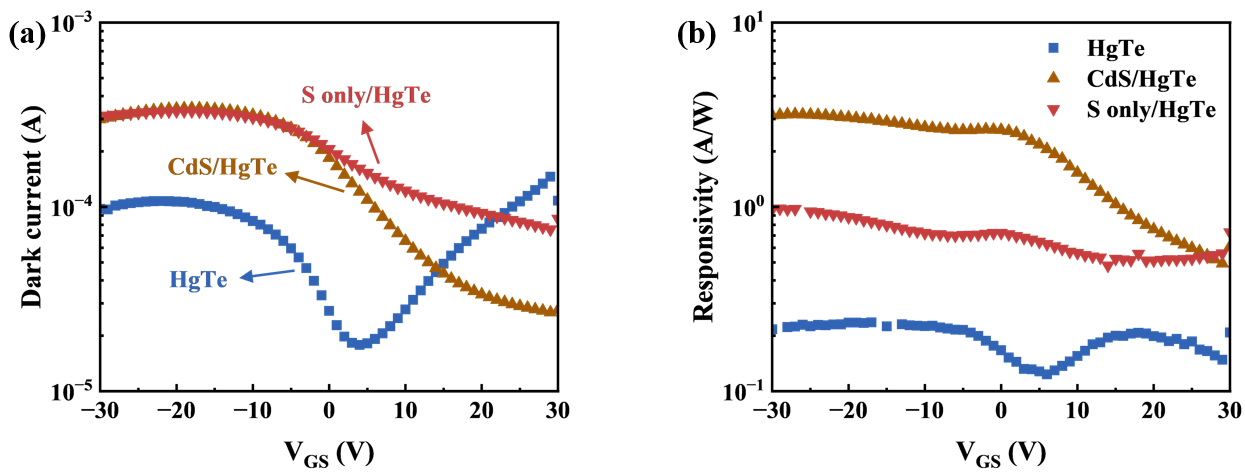


Figure S5. (a) Dark current and (b) responsivity comparison of pristine HgTe, S-only/HgTe, and CdS/HgTe devices


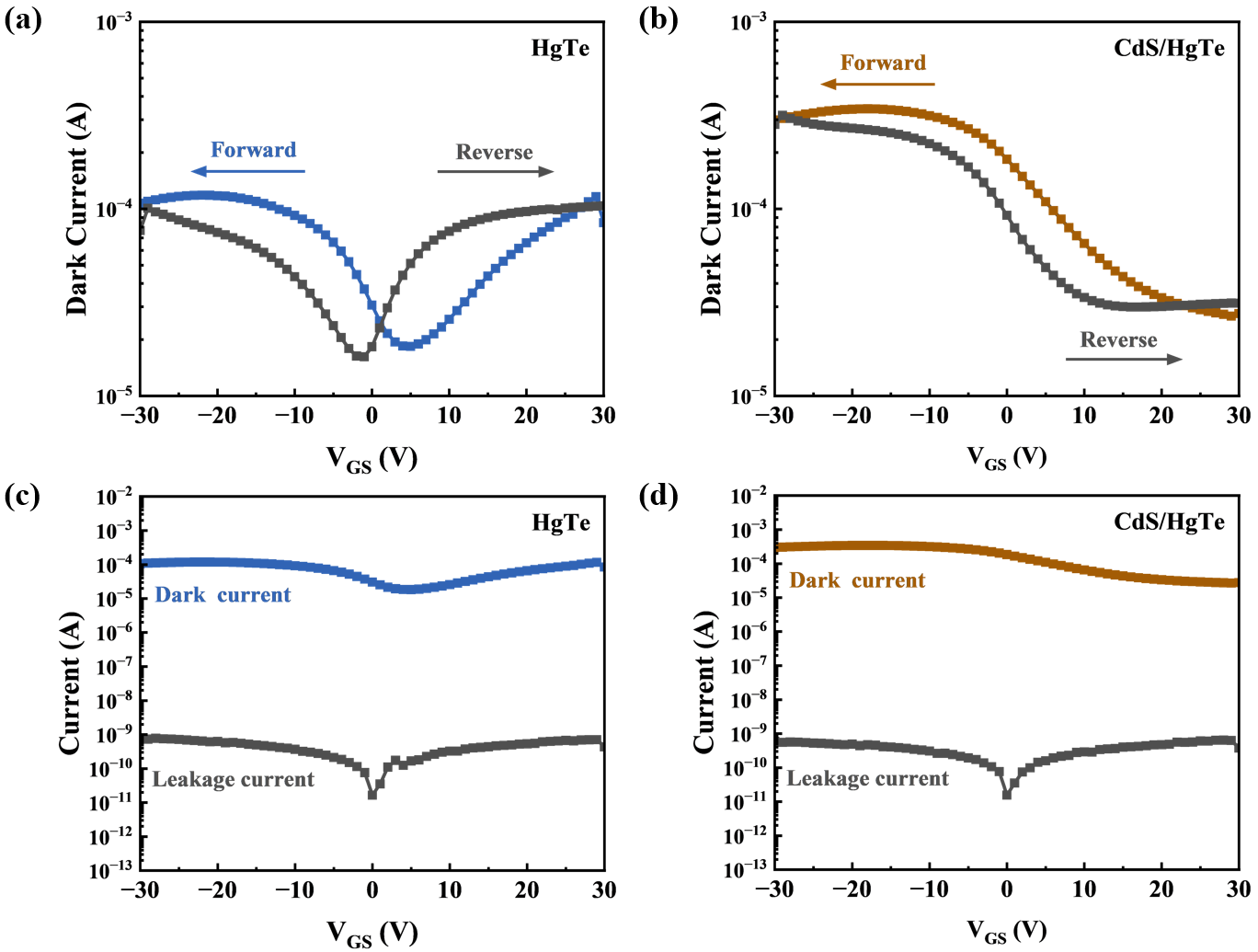
Figure S6. The forward and reverse transfer curves for a) HgTe and b) CdS/HgTe heterojunction phototransistors. Typical transfer characteristics and corresponding gate leakage currents of (c) HgTe and (d) CdS/HgTe heterojunction phototransistors.


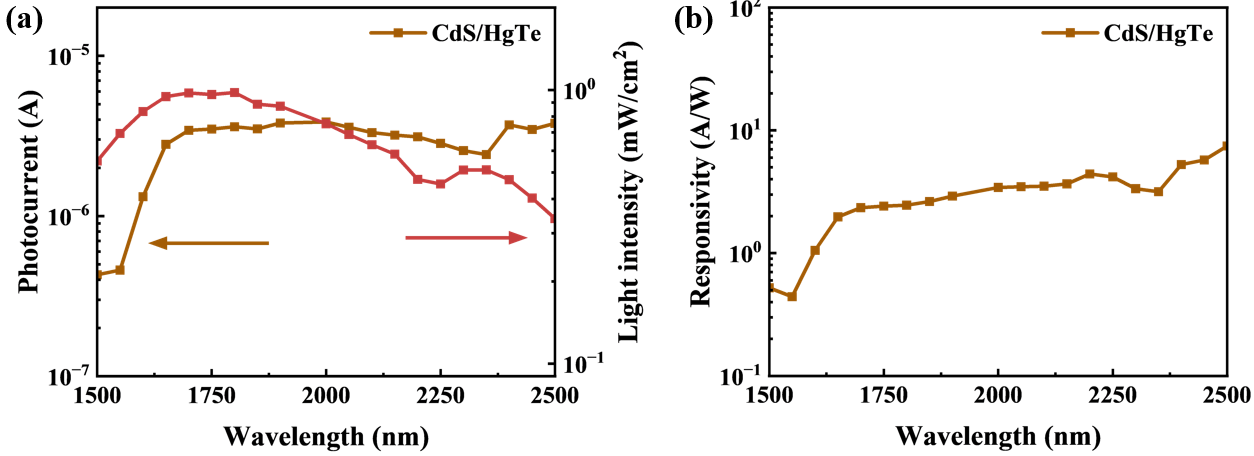


Figure S7. CdS/HgTe phototransistor wavelength-dependent (a) photocurrent and incident light intensity, and (b) responsivity.


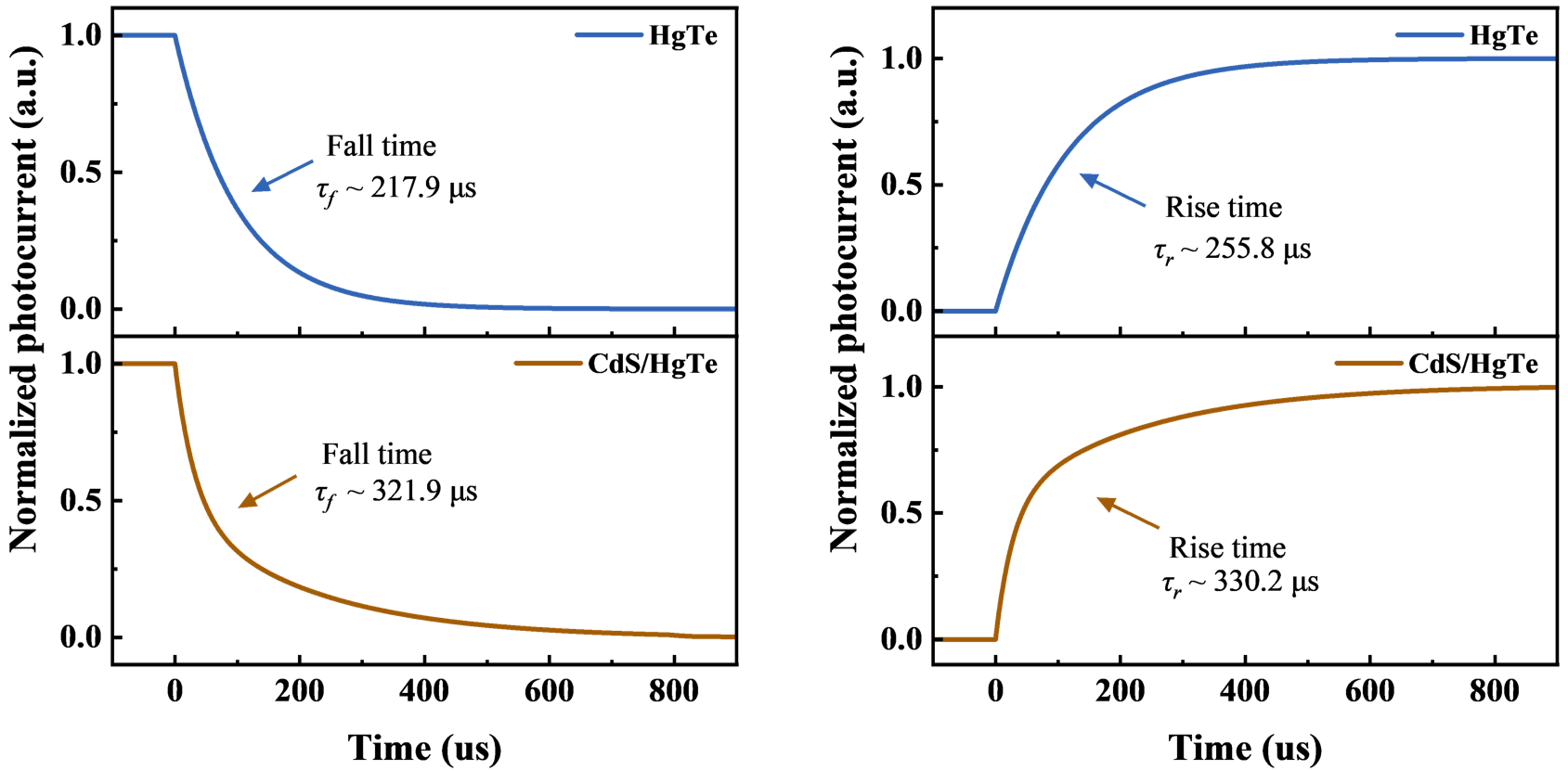


Figure S8. Transient fall and rise photoresponse.
